# Evaluation of Biochemical Characteristics in a Retrospective Cohort of COVID-19 Patients

**DOI:** 10.7759/cureus.58889

**Published:** 2024-04-24

**Authors:** Ahmed Ali Jerah, Abdullah Farasani, Hisham I Abu-Tawil, Hadi Kuriri, Anwar Kuriri, Mansour Alkhayrat, Kholood Kariri, Sami Ali Kariri, Siddig I Abdelwahab

**Affiliations:** 1 Department of Medical Laboratory Technology, Faculty of Applied Medical Sciences, Jazan University, Jazan, SAU; 2 Biomedical Research Unit, Medical Research Center, Jazan University, Jazan, SAU; 3 Department of Laboratory and Blood Bank, Prince Mohammed bin Nasser Hospital, Ministry of Health, Jazan, SAU; 4 Department of Clinical Laboratory and Blood Bank, King Faisal Medical City For Southern Regions, Abha, SAU; 5 Department of Clinical Laboratory and Blood Bank, Samtah General Hospital, Samtah, SAU; 6 Department of Medical Administration, Samtah General Hospital, Samtah, SAU; 7 Department of Nursing Administration, Samtah General Hospital, Samtah, SAU; 8 Department of Pharmacy, Samtah General Hospital, Samtah, SAU

**Keywords:** principal components analysis, retrospective design, saudi arabia, biochemical parameters, covid-19

## Abstract

Background: Coronavirus disease 2019 (COVID-19) has had a significant impact on global health and healthcare systems. This retrospective study aimed to assess the association between biochemical parameters and outcomes in COVID-19 patients in Jazan, Saudi Arabia.

Methods: After establishing the inclusion criteria and obtaining ethical approval, data from 156 reverse transcriptase-polymerase chain reaction (RT-PCR)-confirmed COVID-19 patients were collected from electronic medical records from a general hospital in Samtah, Jazan, from April 2020 to October 2021. The collected data included patient demographics and liver, kidney, heart, and electrolyte function marker levels. Descriptive, inferential, and principal component analyses were conducted.

Results: Survival rates varied according to age and body mass index (BMI). Statistical analysis demonstrated that the levels of aspartate aminotransferase (AST), alanine aminotransferase (ALT), alkaline phosphatase (ALP), sodium (Na), potassium (K), blood urea nitrogen (BUN), creatinine (Cr), creatine kinase (CK), CK myocardial band (MB), and lactate dehydrogenase (LDH) were significantly higher (P < 0.05) than the reference values, as assessed using the one-sample t-test. Principal component analysis (PCA) also revealed an underlying pattern in the variation of these biochemical markers. These findings suggest that certain biochemical parameters may serve as useful indicators for monitoring the condition of COVID-19 patients.

Conclusion: This retrospective study in Jazan, Saudi Arabia highlights the association between biochemical parameters and outcomes in COVID-19 patients. Elevated levels of markers of liver, kidney, heart, and electrolyte function suggest organ damage and dysregulation. The pattern identified through PCA provides insights into disease severity. Monitoring these parameters may serve as valuable indicators for assessing COVID-19 patients. Further research is needed to validate these findings, explore their potential for personalized treatment strategies, and improve patient outcomes during the ongoing pandemic.

## Introduction

The coronavirus disease 2019 (COVID-19) pandemic, caused by the novel coronavirus severe acute respiratory syndrome coronavirus 2 (SARS‑CoV‑2), posed significant global health challenges after its emergence in late 2019. The disease spread rapidly, resulting in millions of cases and substantial morbidity and mortality worldwide [1]. Understanding the clinical and biochemical characteristics of COVID-19 patients is crucial for effective management and improved outcomes. The clinical presentation of COVID-19 varies widely, ranging from asymptomatic or mild respiratory symptoms to severe pneumonia and acute respiratory distress syndrome (ARDS) [2]. Although respiratory symptoms and radiographic findings have been the primary focus of diagnosis and management, recent evidence suggests that biochemical markers can provide valuable insights into disease severity and prognosis [3].

Several studies have reported alterations in various biochemical parameters among COVID-19 patients, including markers of inflammation, coagulation, liver function, renal function, and cardiac injury. Elevated levels of inflammatory markers, such as C-reactive protein (CRP), interleukin (IL)-6, and procalcitonin, have been associated with disease severity and poor outcomes in COVID-19 patients [4,5].

Moreover, abnormalities in coagulation parameters, including D-dimer and fibrinogen levels, have been linked to an increased risk of thromboembolic events and mortality [6,7]. Liver dysfunction, characterized by elevated levels of liver enzymes such as alanine aminotransferase (ALT) and aspartate aminotransferase (AST), has been observed in a subset of COVID-19 patients, indicating potential hepatocellular injury [8,9].

Similarly, acute kidney injury (AKI) has been reported in a significant proportion of severely ill patients, with elevated serum creatinine levels and reduced glomerular filtration rate (GFR) associated with worse outcomes [10,11]. Cardiac injury, as evidenced by elevated cardiac biomarkers such as troponin and brain natriuretic peptide (BNP), has been observed in COVID-19 patients and is associated with an increased risk of mortality [12,13].

This retrospective study aimed to assess the biochemical characteristics of COVID-19 patients in Jazan, Saudi Arabia, and to examine their association with patient outcomes. These findings have the potential to contribute to the existing body of knowledge and aid healthcare professionals in risk stratification, treatment decision-making, and resource allocation.

## Materials and methods

This retrospective study included 156 patients (83 male and 73 female), with a median age of 63.5 years. Data were collected from Samtah General Hospital, Jazan, Saudi Arabia, between April 2020 and October 2021. Demographic information and biochemical parameters of patients were obtained from their electronic medical records. As this study was retrospective in nature, there was no direct contact or potential risk to patients, and their privacy and confidentiality were strictly maintained. The diagnosis of COVID-19 was confirmed through laboratory testing using reverse transcriptase-polymerase chain reaction (RT-PCR) analysis of nasopharyngeal swab specimens, following the guidelines established by the Centers for Disease Control and Prevention, Saudi Arabia.

Variables and indices of observation

The retrospective analysis included variables such as sex, age, weight, height, BMI, mortality, and blood biochemical indices. Patients were divided into two groups based on their outcomes: death and survival. Blood biochemical analyses included several parameters including aspartate aminotransferase (AST), alanine aminotransferase (ALT), alkaline phosphatase (ALP), glucose, sodium (Na), potassium (K), chloride (Cl), blood urea nitrogen (BUN), creatinine, creatine kinase (CK), CK-myocardial band (CK-MB), and lactate dehydrogenase (LDH). The relationship between these blood biochemical indices and patient outcomes was analyzed to understand the impact of these factors on the outcome of COVID-19. Furthermore, the major blood biochemical indices in COVID-19 patients were compared with their reference intervals [14] to assess deviations. Stringent quality control measures were rigorously enforced during laboratory analysis of biochemical parameters to ensure the veracity and dependability of the results obtained. The current study did not include the data for comorbidity. The data was collected at the time of admission. 

Inclusion and exclusion criteria

A total of 2088 individuals who were diagnosed with COVID-19 and screened were included in the study. However, after applying exclusion criteria, such as patients with a stay of less than 24 hours in the ER department, those sent home for recovery or home isolation, and patients admitted to two different admission sites (wards), a preliminary cohort of 217 remaining patients was obtained. The inclusion criteria were as follows: patients diagnosed with COVID-19, admitted to Samtah Hospital, and having a length of stay of one day or more. After applying the inclusion criteria, a final cohort of 156 patients met all requirements and were included in the study.

Statistical analysis

Statistical description and analysis of the data were performed using IBM SPSS Statistics for Windows, Version 20.0 (Released 2011; IBM Corp., Armonk, New York, United States). Continuous variables are presented as medians or simple ranges, depending on their distribution. Categorical variables are summarized as counts and percentages. The normality of the distribution of continuous variables was confirmed using the Kolmogorov-Smirnov test. A one-sample t-test was used to compare the levels of biochemical parameters (mean ± SD) with their respective reference intervals. Statistical significance was set at P < 0.05.

Ethical approval

This study was approved by the Ethical Committee of the Health Directorate in the Jazan Region (approval number: 2364). The study adhered to the ethical guidelines of the Declaration of Helsinki and other relevant regulations. Patient privacy and confidentiality were ensured through data anonymization and secure storage. As a retrospective analysis, patient consent was not required, and there was no direct interaction or potential harm to patients. The research team maintained its integrity and reported the findings while preserving anonymity. Ethical approval ensured the study's compliance with ethical standards and protected the rights and well-being of the patients involved.

## Results

The distribution of biochemical characteristics among a retrospective cohort of COVID-19 patients, categorized by survival and death outcomes, is presented in Table 1. These findings provide important insights into the characteristics of the cohort. BMI was analyzed among the patients, and the majority fell into the overweight and obese categories. Specifically, 14 patients (9.2%) in the underweight category survived, whereas 13 patients (8.5%) in that category died. In the normal BMI range, 56 patients (36.6%) survived and 49 patients (32.0%) died. Among those classified as overweight, 58 patients (37.9%) survived and 50 patients (32.7%) died. In the obese category, 25 patients (16.3%) survived and 18 patients (11.8%) died. Age played a significant role in COVID-19 outcomes. The highest proportion of patients was found in the age group > 60 years. Among patients aged < 40 years, 42 (26.9%) survived and 37 (23.7%) died. In the 40-60 years age group, 30 patients (19.2%) survived and 26 patients (16.7%) died. Among patients aged > 60 years, 84 (53.8%) survived and 70 (44.9%) died.

**Table 1:** Demographic characteristics in a retrospective cohort of COVID-19 patients* *A total of 156 patients with confirmed COVID-19 status were identified through reverse transcription polymerase chain reaction (RT-PCR) testing at Samtah Hospital in Jazan, Saudi Arabia. The data was extracted from the hospital's electronic medical records between April 2020 and October 2021. COVID-19: coronavirus disease 2019

Variables	Overall sample, n (%)	Survivors, n (%)	Non-Survivors, n (%)
BMI (kg/m²)			
Underweight (< 18.5)	14 (9.2)	13 (8.5)	1 (0.7)
Normal (18.5-24.9)	56 (36.6)	49 (32.0)	7 (4.6)
Overweight (25-29.9)	58 (37.9)	50 (32.7)	8 (5.2)
Obese (> 30)	25 (16.3)	18 (11.8)	7 (4.6)
Age (years)			
Less than 40	42 (26.9)	37 (23.7)	5 (3.2)
40-60	30 (19.2)	26 (16.7)	4 (2.6)
More than 60	84 (53.8)	70 (44.9)	14 (9.0)
Total	156 (100)	115 (83.9)	22 (16.1)

Various biochemical markers including AST, ALT, and ALP were examined. Most patients had AST, ALT, and ALP levels within normal ranges (Table 2). However, there were differences between the survivors and non-survivors. For example, among patients with AST levels below 8 IU/L, 10 patients (6.7%) survived. In the range of 8-49 IU/L, 73 patients (49.0%) survived and 12 (8.10%) died. Among patients with AST levels above 49 IU/L, 43 (28.9%) survived and 11 (%) died. Glucose, Na, K, and Cl levels were also analyzed. Elevated glucose levels were observed in many patients, particularly in the non-survivors. Of the patients with glucose levels above 6.5 mmol/L, 84 (58.3%) survived and 18 (12.5%) died. The Na and K levels were normal in both the survivors and non-survivors. Similarly, the chloride levels were generally within the normal range, with no significant differences between the survivors and non-survivors. The BUN and creatinine levels were also assessed. Most patients had BUN and creatinine levels within normal ranges. However, a small proportion of patients had elevated levels, particularly in the non-survivors. Among patients with BUN levels above 6.9 mmol/L, 55 (37.2%) survived and 10 (6.8%) died. For creatinine levels above 90 mmol/L, 54 patients (35.3%) survived and 12 patients (7.8%) died.

**Table 2:** Biochemical characteristics in a retrospective cohort of COVID-19 patients* *A total of 156 patients with confirmed COVID-19 status were identified through reverse transcription polymerase chain reaction (RT-PCR) testing at Samtah Hospital in Jazan, Saudi Arabia. The data was extracted from the hospital's electronic medical records between April 2020 and October 2021. COVID-19: coronavirus disease 2019

Parameters	Overall sample, n (%)	Survivors, n (%)	Non-Survivors, n (%)
AST (IU/L)			
< 8	10 (6.7)	10 (6.7)	0 (0.0)
8-49	85 (57.0)	73 (49.0)	12 (8.1)
> 49	54 (36.21)	43 (28.9)	11 (7.4)
ALT (IU/L)			
< 16	5 (3.4)	5 (3.4)	0 (0.0)
16-43	120 (80.5)	99 (66.4)	21 (14.1)
> 43	24 (16.1)	22 (14.8)	2 (1.3)
ALP (IU/L)			
< 43	7 (4.9)	6 (4.2)	1 (0.7)
43 - 128	95 (66.4)	80 (55.9)	15 (10.5)
> 128	41 (28.7)	34 (23.8)	7 (4.9)
Glucose (mmol/L)			
< 3.4	3 (2.1)	2 (1.4)	1 (0.7)
3.4 – 6.5	39 (27.1)	36 (25.0)	3 (2.1)
> 6.5	102 (70.8)	84 (58.3)	18 (12.5)
Na mmol/L			
< 135	67 (43.8)	54 (35.3)	13 (8.5)
135-142	67 (43.8)	60 (39.2)	7 (4.6)
> 142	19 (12.4)	16 (10.5)	3 (2.0)
Potassium (mmol/L)			
< 3.1	5 (3.3)	4 (2.6)	1 (0.7)
3.1-4.7	117 (77.5)	100 (66.2)	17 (11.3)
> 4.7	29 (19.2)	26 (17.2)	3 (2.0)
CL (mmol/L)			
< 100	42 (43.8)	34 (35.4)	8 (8.3)
100-109	49 (51.0)	46 (47.9)	3 (3.1)
> 109	5 (5.2)	5 (5.2)	0 (0.0)
BUN mmol/L			
< 2.2	9 (6.1)	8 (5.4)	1 (0.7)
2.2-6.9	74 (50.0)	62 (41.9)	12 (8.1)
> 6.9	65 (43.9)	55 (37.2)	10 (6.8)
Creatinine mmol/L			
< 50	25 (16.3)	23 (15.0)	2 (1.3)
50-90	62 (40.5)	53 (34.6)	9 (5.9)
> 90	66 (43.1)	54 (35.3)	12 (7.8)
CK (IU/L)			
< 18	1 (0.8)	1 (0.8)	0 (0.0)
18-137	79 (65.3)	64 (52.9)	15 (12.4)
> 137	41 (33.9)	36 (29.8)	5 (4.1)
CK MB (IU/L)			
< 0.8	1 (0.8)	1 (0.8)	0 (0.0)
0.8-3.9	0 (0)	0 (0.0)	0 (0.0)
> 3.9	119 (99.2)	99 (82.5)	20 (16.7)
LDH (IU/L)			
< 232	37 (27.0)	34 (24.8)	3 (2.2)
232-518	71 (51.8)	56 (40.9)	15 (10.9)
> 518	29 (21.2)	25 (18.2)	4 (2.9)
Total	156 (100)		

Finally, CK, CK-MB, and LDH levels were examined. Most patients had CK, CK-MB, and LDH levels within normal ranges. However, elevated LDH levels were observed in a small proportion of the patients, especially in the non-survivors. Among patients with LDH levels above 518 IU/L, 25 patients (18.2%) survived and four (2.9%) died. These findings provide valuable insights into the distribution of biochemical characteristics among COVID-19 patients and shed light on potential associations with disease outcomes. It is essential to consider these results in the context of the study's limitations and recognize that other factors may also influence COVID-19 severity and mortality.

Table 3 presents the results of the statistical tests comparing the average values of each biochemical parameter to the normal range. Kolmgrov-Smirov test showed the data was normally distributed (p>0.05). The mean difference, 95% confidence interval (CI), and p-values are reported for each parameter. The results indicated that the average AST level showed a significant difference with a mean difference of 39.69 IU/L (95%CI 20.34-59.05; p < 0.001). Similarly, the average ALT level exhibited a significant difference, with a mean difference of 54.72 IU/L (95%CI 1.99-107.44; p = 0.043). The ALP level also displayed a significant difference with a mean difference of 108.92 IU/L (95%CI 43.71-174.14; p = 0.002). Additionally, the average sodium level showed a significant difference with a mean difference of 6.30 mmol/L (95%CI: 0.43-12.19; p = 0.038). The average K level exhibited a significant difference with a mean difference of 0.49 mmol/L (95%CI 0.36-0.62; p < 0.001). Furthermore, the average BUN level showed a significant difference with a mean difference of 7.38 mmol/L (95%CI 5.39-9.37; p < 0.001). The average creatinine level exhibited a significant difference with a mean difference of 132.11 mmol/L (95%CI 84.27-179.96; p < 0.001). Similarly, the average CK level showed a significant difference, with a mean difference of 525.34 IU/L (95%CI 282.72-767.96; p < 0.001). The average CK-MB level displayed a significant difference with a mean difference of 39.59 IU/L (95%CI 13.28-65.90; p = 0.004). Additionally, the average LDH level was significantly different, with a mean difference of 348.48 IU/L (95%CI 150.87-546.09; p = 0.001).

**Table 3:** The results of statistical tests to compare the average of each biochemical parameter to its normal range** *One-Sample Test. The blood biochemical analysis encompassed several parameters, including aspartate aminotransferase (AST), alanine aminotransferase (ALT), alkaline phosphatase (ALP), glucose, sodium (Na), potassium (K), chloride (Cl), blood urea nitrogen (BUN), creatinine, creatine kinase (CK), potassium (K), creatine kinase-MB (CK-MB), and lactate dehydrogenase (LDH). **A total of 156 patients with confirmed COVID-19 status were identified through reverse transcription polymerase chain reaction (RT-PCR) testing at Samtah Hospital in Jazan, Saudi Arabia. The data was extracted from the hospital's electronic medical records between April 2020 and October 2021. The mean differences between the initial and subsequent values were calculated by subtracting the initial value upon admission from the subsequent value for each individual. Then, these differences were used to calculate the mean difference and perform the t-test to assess whether the mean difference was statistically significant.

Parameters	Mean difference	95% CI		p-value*
		Lower	Upper	
AST (IU/L)	39.69	20.34	59.05	0.000
ALT (IU/L)	54.72	1.99	107.44	0.043
ALP (IU/L)	108.92	43.7129	174.1408	0.002
Glucose (mmol/L)	13.31	-0.90	27.53	0.066
Na (mmol/L)	6.30	0.43	12.19	0.038
K (mmol/L)	0.49	0.36	0.62	0.000
Cl (mmol/L)	6.60	-0.45	13.65	0.060
BUN (mmol/L)	7.38	5.39	9.37	0.000
Creatinine (mmol/L)	132.11	84.27	179.96	0.000
CK (IU/L)	525.34	282.72	767.96	0.000
CK-MB (IU/L)	39.59	13.28	65.90	0.004
LDH (IU/L)	348.48	150.87	546.09	0.001

Table 4 presents the results of the PCA-based cluster analysis for COVID-19 phenotypes. The table displays the loadings of each variable (biochemical parameter) on the four identified components. The first component (Component 1) was strongly associated with CK-MB, ALP, and LDH, as indicated by high loadings of 0.950, 0.931, and 0.865, respectively. This component represents a cluster of biochemical parameters related to cardiac markers and liver functions. The second component (Component 2) showed moderate loadings for CK, CK-MB, and ALT, with values of 0.531, 0.557, and 0.923, respectively. This component suggests the presence of a group of parameters associated with muscle damage and liver enzymes. The third component (Component 3) was primarily influenced by Na and Cl, with loadings of 0.960 and 0.886, respectively. This component represents a cluster related to the electrolyte balance. The fourth component (Component 4) exhibited high loadings for AST, BUN, glucose, and creatinine, with values of 0.810, 0.731, 0.689, and 0.636, respectively. This component suggested a cluster associated with liver function, kidney function, and glucose metabolism. The cumulative variance explained by these four components was 74.56%. The Kaiser-Meyer-Olkin measure of sampling adequacy, which assesses the suitability of the data for PCA, has a value of 0.602. It is important to note that the extraction method used was PCA and the rotation method was Varimax with Kaiser Normalization. These methods help identify the most significant components and optimize the interpretability of the results. Overall, these findings from the PCA-based cluster analysis provide insights into the underlying patterns and relationships among biochemical parameters in COVID-19 phenotypes. These can assist in identifying distinct clusters of patients based on their biochemical profiles, which may have implications for disease severity, prognosis, and treatment response.

**Table 4:** COVID-19 phenotypes by principal component analysis-based cluster analysis* Extraction Method: Principal Component Analysis; Rotation Method: Varimax with Kaiser Normalization; Rotated Component Matrix: the cumulative variance explained is 74.56%; Kaiser-Meyer-Olkin Measure of Sampling Adequacy = 0.602. *A total of 156 patients with confirmed COVID-19 status were identified through reverse transcription polymerase chain reaction (RT-PCR) testing at Samtah Hospital in Jazan, Saudi Arabia. The data was extracted from the hospital's electronic medical records between April 2020 and October 2021. In principal component analysis (PCA), a component refers to a new variable created by combining the original variables in a way that captures the most important information and variation in the data. Empty cells in a PCA table indicate that the biomarker does not significantly contribute to that specific component. CK-MB: creatine kinase-myocardial band; ALP: alkaline phosphatase; LDH: lactate dehydrogenase; K: potassium; Na: sodium; Cl: chloride; CK: creatine kinase; ALT: alanine aminotransferase; AST: aspartate aminotransferase; BUN: blood urea nitrogen; COVID-19: coronavirus disease 2019

Parameters	Component 1	Component 2	Component 3	Component 4
CK-MB (U/L)	0.950			
ALP (U/L)	0.931			
LDH (U/L)	0.865			
K (mmol/L)	0.494			
Na (mmol/L)		0.960		
Cl (mmol/L)		0.886		
CK (U/L)	0.531	0.557		
ALT (U/L)			0.923	
AST (U/L)			0.810	
BUN (mmol/L)				0.731
Glucose (mmol/L)				0.689
Creatinine (umol/L)				0.636

## Discussion

In this retrospective study, we aimed to evaluate the effects of biochemical parameters on the outcomes of COVID-19 patients in Jazan, Saudi Arabia. The clinical features and outcomes of COVID-19 patients are complex [15]. Both symptomatic and asymptomatic individuals play a significant role in spreading the infection, primarily through respiratory droplets and close contact via aerosol transmission in enclosed spaces for prolonged periods [16]. Clear guidelines and research on COVID-19 patients are currently lacking [17]. Therefore, it is crucial to focus on the laboratory findings, particularly biochemical parameters, in these patients [5,14]. Biochemical parameters are commonly used as early indicators to monitor metabolic status and organ health. Assessing these parameters in COVID-19 patients is highly important for obtaining valuable information regarding disease outcomes [18].

Biochemical variations in COVID-19 may be attributed to the impact of the virus on multiple organ systems, including the respiratory system, liver, kidneys, and cardiovascular system. Elevated AST, ALT, and ALP levels indicate liver dysfunction, which can result from direct viral invasion or immune-mediated damage. Imbalances in Na and K levels, as well as elevated BUN and creatinine levels, suggest disturbances in the electrolyte balance and potential kidney dysfunction. Increased levels of CK, CK-MB, and LDH indicate muscle and tissue damage, particularly in severe COVID-19 cases [19]. While these variations are not exclusive to COVID-19, their presence in the study population during the pandemic suggests a potential association with viral infections [13]. Further research is needed to establish a direct causal relationship and understand the underlying mechanisms involved.

The distribution of creatinine levels in COVID-19 patients reveals interesting patterns between survivors and non-survivors (Table 2). In the survival group, 43.1% of the patients had creatinine levels above 90 mmol/L. Of the non-survivors, 15.0% had levels below 50 mmol/L, 34.6% had levels within the range of 50-90 mmol/L, and 35.3% had levels above 90 mmol/L. These findings suggest that a significant proportion of COVID-19 patients experienced elevated creatinine levels, indicating potential kidney involvement or dysfunction. This aligns with previous research showing acute kidney injury (AKI) as a known complication in severe COVID-19 cases [20,21]. Elevated creatinine levels may indicate kidney injury or impaired function, which have been associated with worse outcomes [21]. Elevated creatinine levels may indicate a more advanced stage of the disease or the presence of comorbidities that contribute to renal impairment [22]. Severe kidney injury can lead to complications and impact the overall patient prognosis [23]. The results highlight the importance of monitoring kidney function in COVID-19 patients, especially those with elevated creatinine levels. Monitoring kidney function, particularly in patients with elevated creatinine levels, is crucial for early detection and appropriate management. Other factors, such as demographics, comorbidities, and treatment protocols are necessary to fully understand the relationship between creatinine levels and COVID-19 outcomes.

This study reported that 70.8% of the patients who survived had glucose levels above 6.5 mmol/L, while 25.0% of the non-survivors had levels above 6.5 mmol/L (Table 2). These findings suggest that a significant proportion of COVID-19 patients, particularly those who did not survive, exhibited elevated glucose levels. Hyperglycemia, regardless of its underlying cause (whether related to diabetes mellitus or COVID-19-related metabolic derangement), has been associated with worse outcomes in various infectious diseases, including COVID-19. Elevated glucose levels can contribute to inflammation, impair immune responses, and increase vulnerability to complications [24]. Therefore, it is crucial to manage glucose levels and optimize glycemic control in COVID-19 patients, especially those with pre-existing diabetes, as it may play a vital role in improving outcomes [24,25].

Of the survivors, 99.2% had CK-MB levels above 3.9 IU/L (Table 2). Of the patients who did not survive, 82.5% had CK-MB levels above 3.9 IU/L. Elevated CK-MB levels indicate potential cardiac involvement in COVID-19 patients [26]. COVID-19 has been associated with various cardiovascular complications including myocarditis, myocardial injury, and acute cardiac events. Elevated CK-MB levels in the non-survivors suggest a higher likelihood of cardiac damage or injury, contributing to poorer outcomes [27]. Monitoring CK-MB levels in COVID-19 patients, especially those with cardiovascular risk factors, can help identify individuals at higher risk and guide appropriate management strategies. Higher CK-MB levels may indicate a greater degree of heart damage and an increased risk of adverse events [28]. Early recognition and appropriate management of cardiac complications, such as close cardiac monitoring and tailored treatment regimens, are crucial in COVID-19 patients with elevated CK-MB levels.

The current study found that the liver biochemical parameters were significantly higher than the normal reference values (Table 3). Previous research, including a meta-analysis of 25 studies, has also shown a strong correlation between these markers and COVID-19 mortality [19]. Elevated liver enzymes, such as ALT and AST, have been associated with severe COVID-19 cases and increased mortality rates in studies conducted in China and the United Kingdom [29]. Similarly, a study by Zhang et al. in Wuhan, China, reported that abnormal liver function markers such as elevated ALT and AST levels were significantly higher in non-survivors than in survivors [30]. Another study by Bangash et al. conducted in the United Kingdom identified liver injury, indicated by elevated ALT and AST levels, as a potential predictor of mortality in COVID-19 patients [31]. These findings highlight the consistent association between liver biochemical parameters and COVID-19 mortality. Monitoring these parameters can provide valuable insights for clinicians in evaluating the prognosis of COVID-19 patients.

The results presented in Table 4 demonstrate the outcomes of the PCA-based cluster analysis for COVID-19 phenotypes, specifically focusing on the loadings of each variable (biochemical parameters) on the identified components. The cumulative variance explained by these four components (74.56%) suggested that a substantial portion of the variability in the dataset could be attributed to these clusters of biochemical parameters. This supports the relevance and significance of these components in understanding the biochemical profiles and potential phenotypes of COVID-19 patients. Comparing these findings with those of previous studies can provide insight into the consistency and relevance of the identified clusters. In Component 1, the high loadings of CK-MB, ALP, and LDH suggested a strong association between these parameters and cardiac markers, as well as liver function. This finding aligns with previous studies that identified elevated levels of these markers as indicators of cardiac injury and liver dysfunction in severe COVID-19 cases [29,30]. Therefore, Component 1 supports existing evidence regarding the impact of cardiac and liver involvement in COVID-19.

Component 2, with moderate loadings for CK, CK-MB, and ALT, indicated a group of parameters related to muscle damage and liver enzymes. This finding is consistent with previous research that highlighted the association between elevated CK levels and muscle injury in COVID-19 patients [31]. Additionally, elevated ALT levels have been reported as markers of liver injury in severe COVID-19 cases [29,30]. Therefore, Component 2 reinforces our understanding of the relationship between muscle damage, liver enzymes, and COVID-19. Component 3, primarily influenced by Na and Cl, reflects a cluster related to the electrolyte balance. Although the specific association between electrolyte imbalances and COVID-19 outcomes requires further investigation, maintaining proper electrolyte levels is crucial for overall health and organ function. Therefore, Component 3 highlights the importance of monitoring and managing the electrolyte balance in COVID-19 patients.

Component 4 exhibited high loading for AST, BUN, glucose, and creatinine, suggesting a cluster associated with liver function, kidney function, and glucose metabolism. Previous studies have consistently identified elevated AST levels as an indicator of liver injury in severe COVID-19 cases [29,30]. Moreover, increased BUN and creatinine levels have been reported as markers of kidney dysfunction in COVID-19 patients [31]. Elevated glucose levels have also been associated with poor outcomes in COVID-19 patients with diabetes or hyperglycemia [32]. Therefore, Component 4 supports existing knowledge regarding the impact of liver and kidney function, as well as glucose metabolism, on COVID-19 prognosis. In summary, the results of the PCA-based cluster analysis aligned with those of previous studies, providing further evidence of the association between specific biochemical parameters and COVID-19 outcomes. The identified clusters related to cardiac markers, liver function, muscle damage, electrolyte balance, kidney function, and glucose metabolism contribute to our understanding of the complex pathophysiological mechanisms underlying COVID-19 and may have implications for patient management and prognosis assessment. This retrospective study, conducted in Jazan, Saudi Arabia, aimed to evaluate the effects of biochemical parameters on COVID-19 outcomes. However, there are several limitations to consider. The study design is retrospective and based on past records, which may introduce biases and incomplete data. It is a single-center study with a limited sample size, potentially limiting the generalizability of the findings. Confounding factors such as age, comorbidities, and treatment protocols may need to be adequately controlled for. The study primarily examines cross-sectional data, needing more longitudinal information. The observed associations do not imply causation and further research is required. Publication bias and the need for external validation are additional considerations.

## Conclusions

Assessment of biochemical parameters plays a crucial role in effectively managing COVID-19, including predicting the risk and outcomes associated with the disease. Changes in these biochemical factors indicate abnormalities in various tissues and organs, which are indicative of the progression of COVID-19. AST, ALT, ALP, Na, potassium, BUN, creatinine, CK, CK-MB, and LDH were identified as the most predictive parameters for severe COVID-19. Monitoring these biochemical parameters can also help evaluate the dynamic changes occurring in COVID-19 patients. It is important to note that these findings represent associations, not necessarily causal relationships. Further research and larger-scale studies are needed to validate these findings and explore the underlying mechanisms. Additionally, considering other factors, such as patient demographics, comorbidities, and treatment protocols, is necessary to gain a comprehensive understanding of the relationship between glucose levels and COVID-19 outcomes.
